# Ocular outcome and frequency of neurological manifestations in patients with acute posterior multifocal placoid pigment epitheliopathy (APMPPE)

**DOI:** 10.1007/s12348-012-0077-7

**Published:** 2012-05-11

**Authors:** Bettina C. Thomas, Christian Jacobi, Mirjam Korporal, Matthias D. Becker, Brigitte Wildemann, Friederike Mackensen

**Affiliations:** 1 Interdisciplinary Uveitis Centre, Department of Ophthalmology, University of Heidelberg, Im Neuenheimer Feld 400, 69120 Heidelberg, Germany; 2 Department of Neurology, University of Heidelberg, Heidelberg, Germany; 3 Department of Ophthalmology, Triemli Hospital, Zurich, Switzerland

**Keywords:** APMPPE, Ocular outcome, Neurological complications, Cerebral vasculitis

## Abstract

**Purpose:**

The purpose of this study was to describe the visual prognosis as well as the frequency and clinical severity of central nervous system involvement in all acute posterior multifocal placoid pigment epitheliopathy (APMPPE) patients of one centre.

**Methods:**

A retrospective database review of all patients and a prospective clinical, ophthalmological and neurological follow-up, if possible, were conducted.

**Results:**

Eighteen patients with APMPPE were included with a mean follow-up of 17.1 months. Thirteen patients participated in a follow-up exam. Visual acuity improved in 9 of 18 patients to a mean of 0.17 log minimum angle of resolution (MAR) in the worse eye and remained stable in eight patients (mean, 0.03 logMAR). In the majority of patients, the 30° static perimetry improved at follow-up compared to the initial exams. Still, in up to 50 to 60 %, small visual field defects persisted. Overall, 11 patients (61 %) showed neurologic symptoms of varying severity. The most common neurological symptom was headache in nine (50 %) patients. Other symptoms included paraesthesias, psychosis, vertigo, and, as the most severe complication, stroke due to cerebral vasculitis. Fifteen patients were treated with systemic corticosteroids.

**Conclusions:**

Visual prognosis is good in patients with APMPPE, but visual field defects may remain. Neurological signs and symptoms, especially headaches, are frequent in APMPPE and should be taken seriously. Adequate investigations including MRI and CSF examination should be initiated in these patients.

## Introduction

**Fig. 1:**
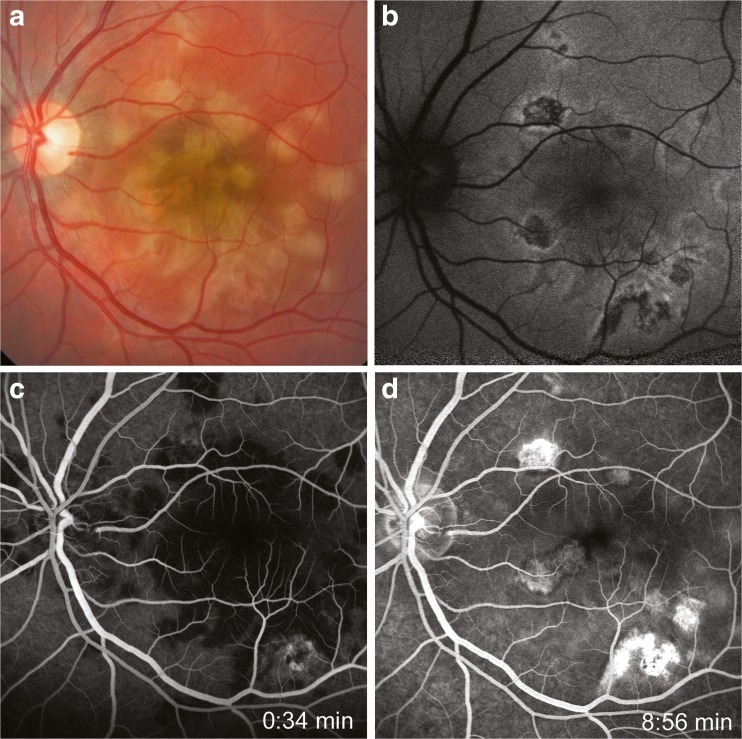
Funduscopically creamy white lesions can be seen (**a**: fundus photography, **b**: autofluorescene image) which later can leave chorioretinal scars. In the fluorescein angiogram, the lesions typically show early hypofluorescence and late hyperfluorescence (**c**, **d**)

Acute posterior multifocal placoid pigment epitheliopathy (APMPPE) is a rare inflammatory eye disease that affects the choriocapillaris, retinal pigment epithelium and outer retina of otherwise healthy young adults. It was first described by Gass in 1968 [1]. Patients present with sudden painless visual loss in one or typically both eyes. Funduscopically creamy white lesions can be seen (Fig. 1a, b) which later can leave chorioretinal scars. In the fluorescein angiogram, the lesions typically show early hypofluorescence and late hyperfluorescence (“blocks early, stains late”, Fig. 1c, d). The full extent of the affected choroid can be seen using indocyanine green as a dye; where the lesions lead to early and late hypofluorescence. Flu-like symptoms often precede the onset of the disease, therefore a viral etiology has been discussed. Several authors described central nervous system (CNS) involvement ranging in severity from headaches to diffuse cerebral vasculitis leading to stroke and death [2]. Overall visual prognosis in APMPPE has been described as good, but there are patients that experience incomplete visual recovery [3]. The purpose of this study is to describe the visual prognosis as well as the frequency and clinical severity of CNS involvement in all APMPPE patients of one centre.

## Methods

A retrospective database review of all APMPPE patients of one centre between 2003 and February 2010 was performed. All patients were asked to participate in a prospective clinical, ophthalmological and neurological follow-up. For the follow-up exam, ophthalmological tests included visual acuity, Amsler test, multifocal electroretinogram (mfERG, RETIscan, Roland Consult), 30° visual field (Octopus 900 or Octopus 101, Haag-Streit International), fundus autofluorescence (FAF) (Heidelberg Retina Angiograph II, Heidelberg Engineering), funduscopy and fundus photography (Zeiss Camera). A fluorescein or indocyanine green angiogram was only performed when clinically indicated (Heidelberg Retina Tomograph II, Heidelberg Engineering). Neurologic tests included a general neurological examination, magnetic resonance imaging (MRI) of the brain, transcranial and carotid duplex (TCD) sonography, electroencephalogram, somatosensory evoked potentials, visual evoked potentials and examination of cerebrospinal fluid (CSF) if clinically indicated. All tests were voluntary for the participants, and refusal of single tests did not lead to exclusion from the study. The study was approved by the ethics committee of the University of Heidelberg. Patients signed a patient consent form.

## Results

Eighteen patients with APMPPE were identified in our database and were included in the study. Thirteen patients (72 %) participated in a follow-up exam; five did not agree to come or could not be contacted. In all patients, there was no doubt that the correct diagnosis was APMPPE. All 18 patients had bilateral involvement. Nine patients were female and nine were male. The mean age at time of diagnosis was 28.61 years (17–58 years). The mean follow-up time was 17.12 months (0–61.43 months).

Visual acuity in the worse eye on the date of presentation ranged between 1.54 and 0 log minimum angle of resolution (MAR) (mean, 0.34 logMAR, Table 1). On last examination, visual acuity was between 0.4 and 0 logMAR (mean, 0.09 logMAR). Taken together, visual acuity improved in 9 of 18 patients from a mean of 0.64 logMAR to a mean of 0.17 logMAR in the worse eye. In nine patients, it remained stable (mean, 0.03 logMAR). Near visual acuity was taken in nine patients (50 %) at follow-up exam and ranged from 0.52 logMAR to 0 logMAR in the worse eye (mean, 0.11 logMAR). All 9 patients (50 %) who were assessed by the Amsler test complained about metamorphopsias in the grid. Of the 13 patients who underwent a follow-up examination, all showed funduscopically small pigmented scars with no activity. The scars could be well visualized using FAF. The results of the fluorescein angiogram (17 patients; one patient refused the procedure) were all in agreement with the diagnosis of APMPPE. The angiograms that were taken in the acute phase all showed the typical staining pattern of early hypofluorescence and late hyperfluorescence. Angiograms that were taken later in the course of the disease showed inactive staining of scars. All 11 multifocal ERGs that were performed (ten at follow-up and one at a relatively acute phase) were pathological: mainly reduced sum responses and or reduced responses of the foveal and parafoveal regions. Fifteen out of 18 patients received a 30° static perimetry. These patients could be divided into three groups: group 1 (three patients) who only had perimetry at one of their first visits, group 2 (six patients) who had perimetry only at follow-up and group 3 (six patients) who had perimetry at one of their first visits as well as at follow-up. To be able to objectively compare the results of the perimetry, the mean defect (MD) values in decibels were analysed. In group 1, the mean MD was 1.1 dB (range, −0.6 to 4.1 dB). In group 2, the mean MD was 0.03 dB (range, −1.6 to 1.3 dB). The exact numbers are displayed in Table 1. In group 3, the mean MD was 4.14 dB (range, −1.2 to 10.8 dB) initially and improved to a mean of 1.77 dB (range, −1.8 to 7.6 dB), indicating that the visual fields improve after the acute phase of the disease. As can be seen in Table 1, all patients but one (patient 8) had an improvement of the visual fields at follow-up compared to the initial exam. One explanation why patient 8 did not improve is that he was our oldest patient and that his general health status at the day of the follow-up exam was poor. Especially in group 3, the MD shows a very broad range confirming that the disease can have varying extents. When looking at the visual fields of all patients that had a follow-up perimetry (i.e. groups 2 and 3), we felt that 6 out of 12 patients had remaining mostly small visual field defects. All but one patient had no more relapses of ocular disease at the end of the first treatment period. The one patient who relapsed had increasing visual field defects that improved after increasing the corticosteroid dosage.

**Table 1:** Ocular follow-up

Patient no.	Sex	Age at DOP (years)	f/u time (weeks)	V/A DOP logMar (R/L)	V/A last visit logMar (R/L)	V/A improv.	Near V/A at f/u logMar (R/L)	Autofluo at f/u	Amsler test	mfERG at f/u	30° perimetry MD in db (R/L), group
1	M	24	1	0/0	0/0	No	nd	No f/u	nd	No f/u	nd
2	F	24	4	0/1.54	0/0.3	Yes	nd	No f/u	nd	No f/u	1.9/4.1 *1*
3	F	35	42.57	0/0.1	0/0	Yes	0/0.1	Scars	R > L metas	nd	1.0/0.4 *2*
4	F	21	105	0/0	0/0	No	nd	Scars	nd	nd	1.0/1.3 *2*
5	M	31	55.14	0.1/0	0.1/0.1	No	nd	Scars	nd	Pathol	−1.6/–1.3 *2*
6	F	28	149.14	0/0.1	0/0	Yes	nd	Scars	nd	Pathol	7.5/10.8 → 3.0/3.4 *3*
7	F	17	82.57	0/0	0/0	No	nd	Scars	nd	Pathol	0.7/0.8 *2*
8	M	58	85.71	1.3/0.9	0.2/0.4	Yes	0.15/0.52	Scars	R/L metas	Pathol	5.7/6.9 → 7.0/7.6 *3*
9	F	38	1.57	0.2/0	0.2/0	No	nd	No f/u	nd	No f/u	nd
10	M	32	263.29	0/0	0/0	No	0.1/0.1	Scars	R/L metas	Pathol	2.5/1.9 → 0.1/0.5 *3*
11	F	18	250	0.1/0	0/0	Yes	0/0	Scars	R/L metas	Pathol	−0.7/–0.8 *2*
12	M	34	1.57	0.3/0.6	0.2/0.3	Yes	nd	No f/u	nd	No f/u	nd
13	F	16	114.57	0/0.9	0/0.1	Yes	0/0.1	Scars	L > R scotoma	R norm, L pathol	0.1/–0.2 *2*
14	M	17	27.43	0/0.7	0/0.3	Yes	nd	No f/u	nd	No f/u	−0.4/1.4 *1*
15	M	28	59	0/0	0/0	No	0/0	Scars	R metas	Pathol	0.7/–0.7 → 0.7/–0.4 *3*
16	M	23	47.29	0/0	0/0	No	0/0	Scars	R > L metas	R pathol; L norm	0.2/–1.2 → −1.6/–1.8 *3*
17	F	50	30.86	0.4/0.1	0/0	Yes	0.2/0.2	Scars	R/L metas	Pathol^a^	6.7/8.7 → 1.3/1.4 *3*
18	M	21	0	0/0	0/0	No	0/0	Scars	L > R metas	L > R pathol	0.2/–0.6 *1*

Italicized numbers after the MD values represent the patient group as described in the text

*DOP* date of presentation, *nd* not done, *f/u* follow-up, *metas* metamorphopsias, *pathol* pathological, *MD* mean defect

^a^mfERG was performed only 1 week after first presentation

Fifteen patients (83 %) were treated with systemic corticosteroids. The initial dosage ranged from 50 to 250 mg (mean, 106 mg) and was determined by the physician on duty who had the first contact with the patient. As far as it can be said retrospectively, the initial dosage was chosen independently of the presence of neurological symptoms. The treatment period ranged between 3 weeks and several months, whereas 47 % of the treated patients received treatment for approximately 1 month. The speed and amount of steroid taper were adjusted to the patient's individual visual and physical recovery (e.g. headaches resolved). The one relapsing patient received additional immunosuppressive medication for 38 weeks which then could be stopped without further relapse. The stroke patient received additional cyclophosphamide for CNS vasculitis.

Eleven patients (61 %) had neurologic symptoms of varying severity (Table 2). In most patients, the neurological symptoms started at the same time or close to the beginning of the ocular symptoms. However, the most severe neurological complication we witnessed—cerebral stroke with hemiparesis and aphasia—took place 2 months after the first symptoms (headaches and visual complaints). This 58-year-old patient (Table 2, patient 8) received 200 mg prednisone at presentation which was slowly tapered. He was on 80 mg prednisone per day when the stroke took place. Six days earlier, he had still complained about headaches. The MRI showed multiple areas of infarction consistent with vasculitis. Embolising cardiac disease, stenosis of cerebral vessels and paraneoplastic disease were ruled out as underlying causes of the stroke. A biopsy of the dura mater revealed lymphocytic infiltrates as a morphological sign of vasculitis.

**Table 2:** Neurological symptoms

Patient no.	Sex	Age at DOP (years)	Neurological symptoms	MRI	Lumbar puncture
1	M	24	Headaches, psychosis	Normal	nd
2	F	24	Headaches	Normal	nd
3	F	35	Headaches	nd	nd
4	F	21	Headaches	Normal	nd
5	M	31	Headaches	Normal	Abnormal
6	F	28	Headaches, myalgia, vertigo, paraesthesias both extremities	Normal	Abnormal
7	F	17	Headaches	Normal	Normal
8	M	58	Initially: headaches; later: stroke, hemiparesis, aphasia	Initially: contrast medium enhancement of optic nerve sheaths and nodular changes in the orbits, possibly granulomatous bilaterally; later: multiple areas of infarction, consistent with vasculitis	Initially: normal; later: abnormal
9	F	38	None	nd	nd
10	M	32	Hypaesthesia, right thumb	Single small unspecific gliotic changes, left hemisphere	nd
11	F	18	None	Normal	nd
12	M	34	None	nd	nd
13	F	16	None	Unspecific bilateral signal intensities in the subcortical frontal area, possibly gliosis	Recommended
14	M	17	None	nd	nd
15	M	28	Paraesthesias, right foot; hypaesthesia, right side of body	Normal	Normal
16	M	23	None	Consistent with right temporal small cavernoma pineal cyst	Normal
17	F	50	None	Normal	nd
18	M	21	Headaches	Normal	nd

*DOP* date of presentation, *nd* not done, *MRI* magnetic resonance imaging

The most common neurological symptom was headache occurring in nine (50 %) patients (Table 2). Three patients had sensory symptoms like hyp- or paraesthesias, one patient experienced psychosis and one patient complained about vertigo. Overall, 12 out of the 14 patients that had a brain MRI did not show signs of vasculitis. Four patients showed abnormalities in the MRI (Table 2, patients 8, 10, 13 and 16). The findings in patients 10 (gliotic changes) and 16 (pineal cyst) were considered insignificant. In patient 16, a CSF examination was performed due to abnormal TCD results, but CSF results were normal. Patient 8, the stroke patient, presented with bilateral contrast medium enhancement of optic nerve sheaths and nodular changes in the orbits, possibly granulomatous before he developed the stroke. CSF examination was normal at this point in time. For patient 13, who had unspecific bilateral signal intensities in the subcortical frontal area, possibly gliosis, a CSF examination was recommended but was declined by the patient.

In total, CSF examination was performed in six patients. Four had normal results. Two patients with headaches and normal MRI showed pleocytosis and/or oligoclonal bands. TCD sonography showed abnormalities in 6 of 14 tested patients. Interestingly, the MRI results, TCD and the CSF analysis did not necessarily correlate with each other. Electrophysiologic exams were not helpful in indicating CNS affection. More detailed results of neurological manifestations and technical abnormalities will be published separately.

## Discussion

APMPPE is a rare disease, and therefore, there are not many systematic reports in the literature. So far, mainly case reports but also some studies with case series have been published. Most studies were done retrospectively as this is often the case with rare diseases. The published studies are difficult to compare and to summarize because emphasis was put on different aspects of the disease. In our study, we focused on two aspects of the disease: the visual, overall outcome and CNS involvement. Seventy-two percent of our patients were examined in a prospective follow-up exam.

Visual loss is the most obvious symptom for the patient and brings the patient to the ophthalmologist. Visual recovery is the most important aim for the patient and the ophthalmologist. In our group of patients, the mean finally measured visual acuity was 0.09 logMAR. Half of the patients had an improvement of visual acuity in the worse eye (mean final visual acuity, 0.17 logMAR) compared to the date of presentation (mean, 0.64 logMar). The other half remained stable (mean first and final visual acuity, 0.03 logMar) but had an overall better initial visual acuity. That visual recovery may be incomplete in some patients is in agreement with Fiore et al. [3]. They found in an extensive literature review as well as in a group of their own patients that APMPPE has a relatively benign visual prognosis, but compared to other white dot syndromes, there are patients with incomplete visual recovery. Fiore states, in agreement with other relatively recent reports [4, 5], that nearly 50 % of the eyes reached an incomplete recovery in visual acuity (≤0.1 logMAR) and that approximately 25 % of the eyes have a more limited visual recovery (≤0.3 logMAR). In contrast, some older publications describe a good visual prognosis [6-9]. A long-term study (average follow-up 5 years) conducted by Gass [10] revealed that only 2 out of 59 eyes achieved a visual acuity less than 0.18 logMAR. Our study also revealed a good outcome looking at visual acuities, but all patients showed scarring of the posterior pole that was mirrored by persistent visual field defects in the central 30° in two thirds of the patients examined. Still, good reading abilities were shown in those patients where near distance visual acuities were obtained, albeit with metamorphopsias in all of these patients. As APMPPE is a disease that afflicts relatively young patients, we need to find out how we can prevent the scarring and thus improve outcome.

Neurological involvement may not be as obvious as the reduction of visual acuity. We observed mainly headaches which occurred in half of the patients. Of patients with headaches, only four described flu-like symptoms. Headaches can represent a neurological symptom on its own or can be part of the flu-like symptoms which patients sometimes report. The most severe neurological complication we witnessed was stroke caused by histologically proven cerebral vasculitis. In a Medline search, we found 31 APMPPE patients with neurological manifestations [2, 3, 11–18]. Cerebral stroke occurred in 16 cases. Our case series adds another 11 patients with neurological symptoms and 1 with cerebral stroke. Several authors describe angiography findings that are in line with cerebral vasculitis (e.g. focal stenosis, lumen fluctuation), but in only two cases, vasculitis was histologically proven [19–21].

Other neurological manifestations that are described in the literature are vertigo, dysarthria, ataxia, tremor, paraesthesias, migraine and cavernous sinus thrombosis [14]. Of note in our study was the fact that MRI results did not necessarily correlate with CSF abnormalities. Patients 5 and 6 (Table 2) had normal MRIs but abnormal CSF findings. Vice versa, our stroke patient (patient 8) displayed pathological findings at cranial MRI, yet had a normal CSF. Pathological CSF results in APMPPE patients with or without major neurological complications have been described [22–24].

We did not detect a clear correlation between ophthalmologic impairment and the rather heterogeneous neurologic manifestations. For example, patient 8, the patient with the worst neurological complication, also had one of the worst visual acuities and MD values. In this case, there is a positive correlation. On the other hand, patient 5 who had headaches and changes in the CSF had a very good visual acuity (V/A) and visual field. Ophthalmologic findings in patient 6 illustrate that even visual acuity and visual field do not necessarily correlate. This patient who complained of different neurological symptoms and had changes in the CSF had a very good V/A, but pronounced field defects. Depending on the localisation of the retinal lesions, especially the involvement of the fovea, patients might have a good V/A, but a bad visual field. To judge if a patient is in danger of neurological complications, the severity of the visual loss and field defects are not a reliable marker. We think that it is more helpful to explicitly ask the patient about neurological symptoms and in the case of doubt lean towards lumbar puncture to rule out CNS involvement. Other non-invasive neurological examinations may be superior (manuscript in preparation).

We conclude that neurological signs and symptoms, especially headaches, are frequent in APMPPE and should be taken seriously. Adequate investigations including MRI and CSF examination should be initiated in these patients. Our patients with persisting neurological symptoms and suspect MRI or CSF results were monitored closely by us and the neurologists. It may be discussed that CSF investigations should be performed even in an asymptomatic, MRI negative APMPPE patient as ocular symptoms may precede neurologic symptoms or ophthalmic treatment may masquerade neurologic manifestation. If neurological symptoms persist despite treatment, it might even be advisory to repeat MRI or CSF as we learn from our stroke patient where the initial MRI and CSF were not able to identify vasculitis. Immunomodulatory treatment might be of prognostic relevance, but no prospective studies have been performed. It is unlikely that they will ever be performed, as the disease is so rare. In our cohort, only three patients did not receive treatment, and their outcome did not differ from the rest of the group. At the same time, 80 mg of prednisone did not prevent the stroke in the described patient. Some authors question the need to treat APMPPE patients arguing that therapy does not alter the natural course of the disease [10, 18, 25]. Fiore et al. [3] conclude that the use of oral corticosteroids remains unclear and that more definitive data are required. Several authors agree that rapid treatment is indicated when CNS complications accompany APMPPE [2, 22].

We think that the very high corticosteroid starting dosages (>1 mg/kg body weight) which were chosen in some of the patients by the physicians on duty for the ophthalmologic aspect alone were too high. But, if one considers the assumed pathogenesis, an immune hyperreactivity after a viral infection, it feels like a logical consequence to use oral corticosteroids to soften that immune response. At the same time, it may prolong ongoing viral activity.

In conclusion, we have shown in this case series of 18 patients that visual prognosis is good in patients with APMPPE, but visual field defects may remain, significantly reducing quality of vision in the majority of the patients. Neurological complications may be serious and have to be considered at the time of diagnosis of APMPPE. Concluding from our results, all patients with the diagnosis APMPPE should receive a clinical neurological examination, cerebral MRI and TCD with subsequent lumbar puncture if any of these three shows abnormalities. Treatment with oral corticosteroids should be discussed with the neurologists and with the patient as a benefit over natural history cannot be guaranteed.
